# Tacrolimus Does Not Affect Early Wound Healing in a Rodent Model of Bowel Anastomoses and Abdominal Wall Closure

**DOI:** 10.1371/journal.pone.0076348

**Published:** 2013-09-26

**Authors:** Martine C. M. Willems, J. Adam van der Vliet, Roger M. L. M. Lomme, Thijs Hendriks

**Affiliations:** Department of Surgery, Division of Vascular and Transplantation Surgery, Radboud University Medical Centre, Nijmegen, The Netherlands; University of Catania, Italy

## Abstract

**Background:**

Use of immunosuppressant drugs has been associated with complications in wound healing. The calcineurin inhibitor tacrolimus is thought to have a relatively low complication rate, but preclinical research has yielded contradictory data, prompting the current comprehensive study

**Methods:**

Three groups of 33 male Wistar rats received a daily subcutaneous dose of 0,5, 2 or 5 mg/kg tacrolimus. A control group received saline. On day 0 a resection of 1 cm ileum and 1 cm colon was performed, and end-to-end anastomoses were constructed. Ten rats of each group were killed on day 3 and day 5 and the remaining animals on day 7. Both anastomoses and the wound in the abdominal wall were analyzed. Wound strength was the primary outcome parameter.

**Results:**

Mean strength of the abdominal wall increased significantly over time in all groups (p<0.0001). Both the breaking strength and the bursting pressure of the ileum and colon anastomoses followed the same pattern. No differences were observed between control and experimental groups. In addition, no consistent differences were found between groups regarding wound hydroxyproline content and the activities of matrix metalloproteinase-2 and -9.

**Conclusion:**

Tacrolimus does not affect early wound healing.

## Introduction

Under different immunosuppressant regimes, wound healing disturbances are seen in 7-53% of kidney transplant recipients [1-4]. The premise that all immunosuppressant drugs have a negative effect on wound healing is widely accepted, although often scientific proof is lacking or inadequate. Clinical studies almost invariably use a regime of several immunosuppressant drugs, making it difficult to attribute adverse effects to a single component [4,5]. Thus, preclinical research is necessary to elucidate potential effects of individual drugs. This way we have demonstrated that everolimus, a m-TOR inhibitor, has a negative, dose- and time-dependent effect on experimental wounds in intestine and abdominal fascia [6,7].

Today, the typical standard regime of immunosuppressant drugs in solid organ transplantation consists of a calcineurin inhibitor such as cyclosporin or tacrolimus, an antiproliferative agent (azathioprine or mycophenolate mofetil) and a steroid. In this regime, tacrolimus is the newer agent, and extensive research has been carried out to establish its benefit over cyclosporine [1,8-10]. In contrast to most immunosuppressive drugs, tacrolimus, a macrolide derived from the fungus *S* *tsukubaensis* , is believed to have few or none adverse effects on wound healing. Still, as a calcineurin inhibitor, tacrolimus affects the first phase of T-cell activation. Because inflammatory T cells play a role in wound healing an effect of tacrolimus on wound healing is conceivable. Reported effects of tacrolimus in preclinical studies are contradictory, ranging from stimulation to inhibition of soft tissue repair [11-16]. A common drawback to these studies is the fact that, almost without exception, only one post-operative time point is evaluated, while wound healing is a complex process with different and overlapping phases.

The present comprehensive study has been performed to evaluate the effects of tacrolimus, used as a single drug in three different doses, and at multiple time points during healing of bowel anastomoses and abdominal fascia in rats.

## Materials and Methods

One hundred thirty-two male Wistar rats (body weight 240-260 g; Harlan, Horst, The Netherlands) were randomly divided into four groups of thirty-three animals. The animals were housed two per cage and allowed to become accustomed to laboratory conditions for one week before the start of the experiment. All animals had free access to water and standard rodent chow (Ssniff Specialdiäten GmbH, Soest, Germany). Three groups received tacrolimus (Prograft®, Astellas, Killorglin, Ireland) subcutaneously in daily dosages of 0.5 (group T0.5), 2.0 (group T2) and 5.0 (group T5) mg/kg/day from the day of operation until the end of the experiment. A control group received saline subcutaneously.

### Ethic Statement

This study was carried out in strict accordance with the National Dutch Act on Experimental Animals. The protocol (80320) was approved by the Animal Ethics Review Committee of the Radboud University, Nijmegen (Permit number DEC-2008-114). In order to minimize suffering all surgery was performed under general anesthesia using isoflurane 3%, in a mixture of oxygen and nitrous oxide. Postoperative analgesia was performed with buprenorphine, 0.02 mg/kg subcutaneously, twice daily for two days. The animals were killed by CO2/CO asphyxiation to reduce as much stress as possible. An individual animal welfare logbook was kept and reported back to the Animal Ethics Review Committee.

### Surgical Procedure

On day 0, a midline laparotomy was performed and followed by a resection of 1 cm ileum 15 cm proximal to the ileocaecal junction and 1 cm colon 3 cm proximal to the rectal peritoneal reflection. End-to-end anastomoses were constructed with eight single-layer, inverting, interrupted 8-0 ethilon (Ethicon) sutures. The abdominal fascia was closed with an absorbable, polygalactin 3-0 suture, the skin was closed with staples. A heating pad was used to maintain body temperature at 38°C. The intestines were covered with gauze pads soaked with 0.9% NaCl to minimize desiccation. Fluid loss was compensated by administering 10 ml of 0.9% subcutaneously direct postoperative. The animals were weighed daily and observed for signs of illness. All operative procedures were performed by the same investigator (MW).

### Wound strength

Ten rats per group were killed on day 3 and day 5 each, and the remaining animals were killed on day 7. In the latter group, EDTA whole blood was sampled for tacrolimus assay (see below). Relaparotomy was performed by excision of a part of the abdominal wall of approximately 4 by 4 cm, including the suture line of the fascia. The anastomoses of ileum and colon were resected with adjacent bowel of approximately 4 cm in length and the suture line in the middle. The intestinal segments were carefully resected, including surrounding tissues and adhesions, and washed in saline. Bursting pressure and breaking strength were measured in the same segment as described previously [17]. In the abdominal wall the breaking strength was measured in the same way; from each segment of the abdominal wall, two separate strips of 1 by 2 cm were collected, with the suture line in the middle, and the breaking strength was measured in both. After biomechanical analysis, segments were cleaned from adhering tissue and standard sized samples containing the suture line were frozen in liquid nitrogen and stored at -80°C until further processing.

### Biochemical analysis and histology

After lyophilisation, tissue samples were weighed, pulverized, and lyophilized again. Both hydroxyproline content and gelatinase activity were measured in control, T2 and T5 groups. The hydroxyproline content, as a measure of the collagen content, was measured by high-performance liquid chromatography after hydrolysis with 6-N-hydrochloric acid and derivatization with dabsyl-chloride.

Preparation of tissue extracts and procedures for gelatin zymography have been described previously [18]. The protein concentration of the extracts was measured using the bicinchoninic acid reagent. The various gelatinase activities were quantitated on the basis of lysed area and expressed as arbitrary units. Comparison of values obtained on different gels was performed by using collagenase type I (from *Clostridium histolyticum*; Sigma Chemical) as an internal standard. The presence of true matrix metalloproteinase (MMP) activity was confirmed by adding 10 mmol/L EDTA or 1,10 phenanthrolene to the buffers used after electrophoresis. Tacrolimus in whole blood was assayed using a PRO-Trac^TM^ ELISA kit from DiaSorin (Still water, Minnesota, USA). Sections of anastomoses originating from separate animals in the groups sacrificed after 7 days that had not been subjected to strength measurements were washed in 0.9% NaCl, spread out, and fixed immediately in a 4% phosphate-buffered formaldehyde solution. Subsequently, the samples were dehydrated and embedded in paraffin blocks. Sections of 4 mm in thickness were stained with hematoxylin and eosin (H&E).

### Statistical analysis

To analyze differences in body weight and MMP-activity a Kruskal Wallis followed by Dunn’s test was used. Data of breaking strength, bursting pressure and hydroxyproline content were analysed with ANOVA followed by Tukey-Kramer post test.

## Results

The mean trough level of tacrolimus, as measured in whole blood collected on day 7 after operation, was 0.3 ± 0.2 (SD) ng/ml in the control group and 4.9 ± 2.4, 10.1 ± 2.0 and 12.3 ± 5.6 ng/ml, respectively, in the T0.5, T2 and T5 groups.

Eight animals died prematurely or were taken out of the experiment because of poor health: one each in the control and T0.5 groups (ileus), two in the T2 group (unknown reasons and excessive weight loss, respectively) and four in the T5 group (two after excessive weight loss and two of unknown reasons). Diarrhea, observed at least once during the experiment, was found in one thirty-second of the surviving rats in the control group and in 0/32, 2/31 and 7/29 (p=0.022) in the T0.5, T2 and T5 groups, respectively.

**Figure 1:**
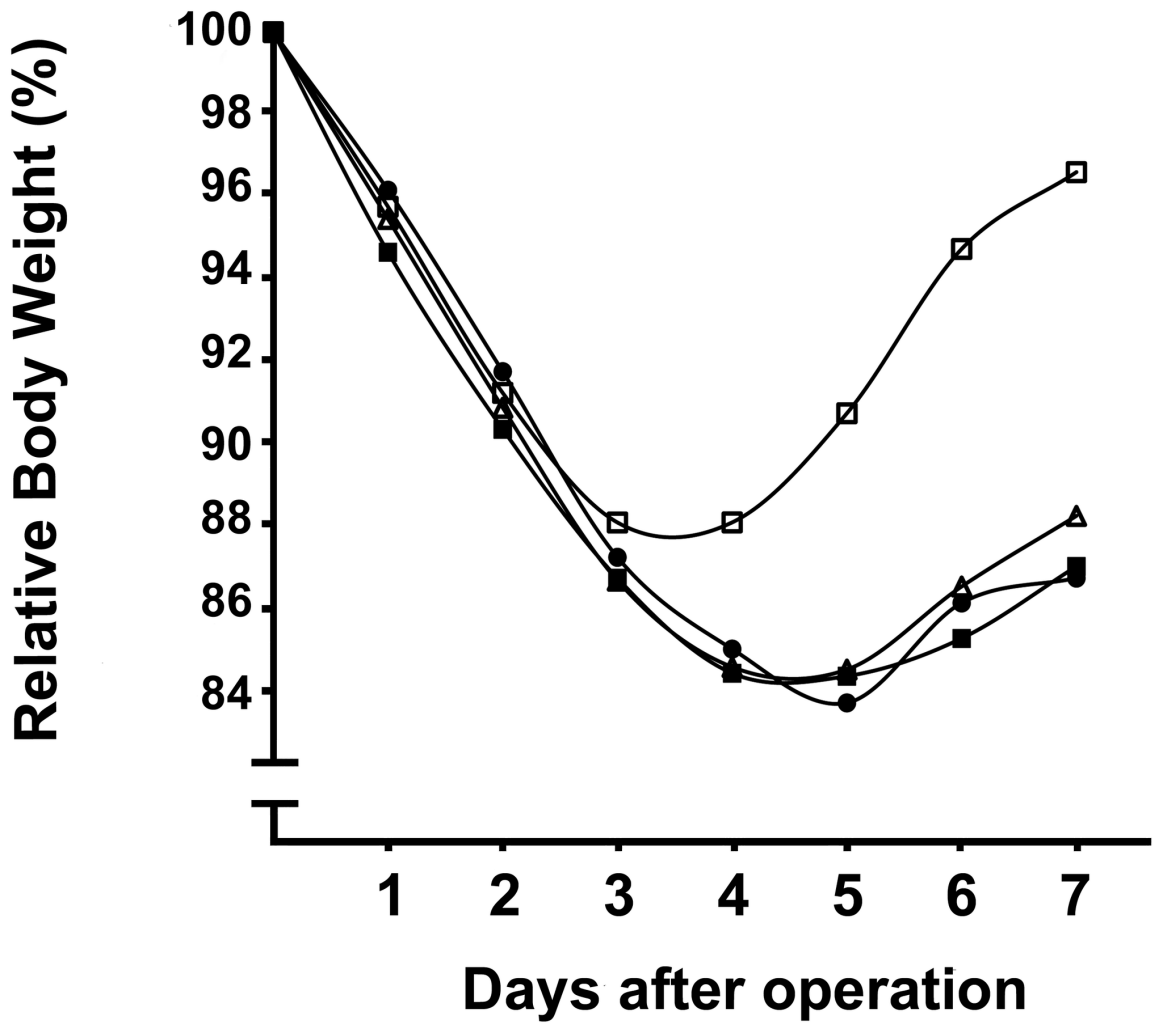
Postoperative course of body weight. Points represent average relative body weight, in relation to the weight prior to operation, for the control group (□) and the groups receiving tacrolimus: T0.5 (◼), T2 (Δ) and T5 (●).

All animals experienced a transient weight loss of approximately 10-15% of their body weight. From day 4 onwards they regained weight, those in the control group approximating their pre-operative weight at day 7 (Figure 1). However, the weight gain in animals receiving tacrolimus was less than in the control group. The relative weight in all experimental groups at day 7 was significantly (p<0.05) lower than in the control group.

### Wound strength

**Figure 2:**
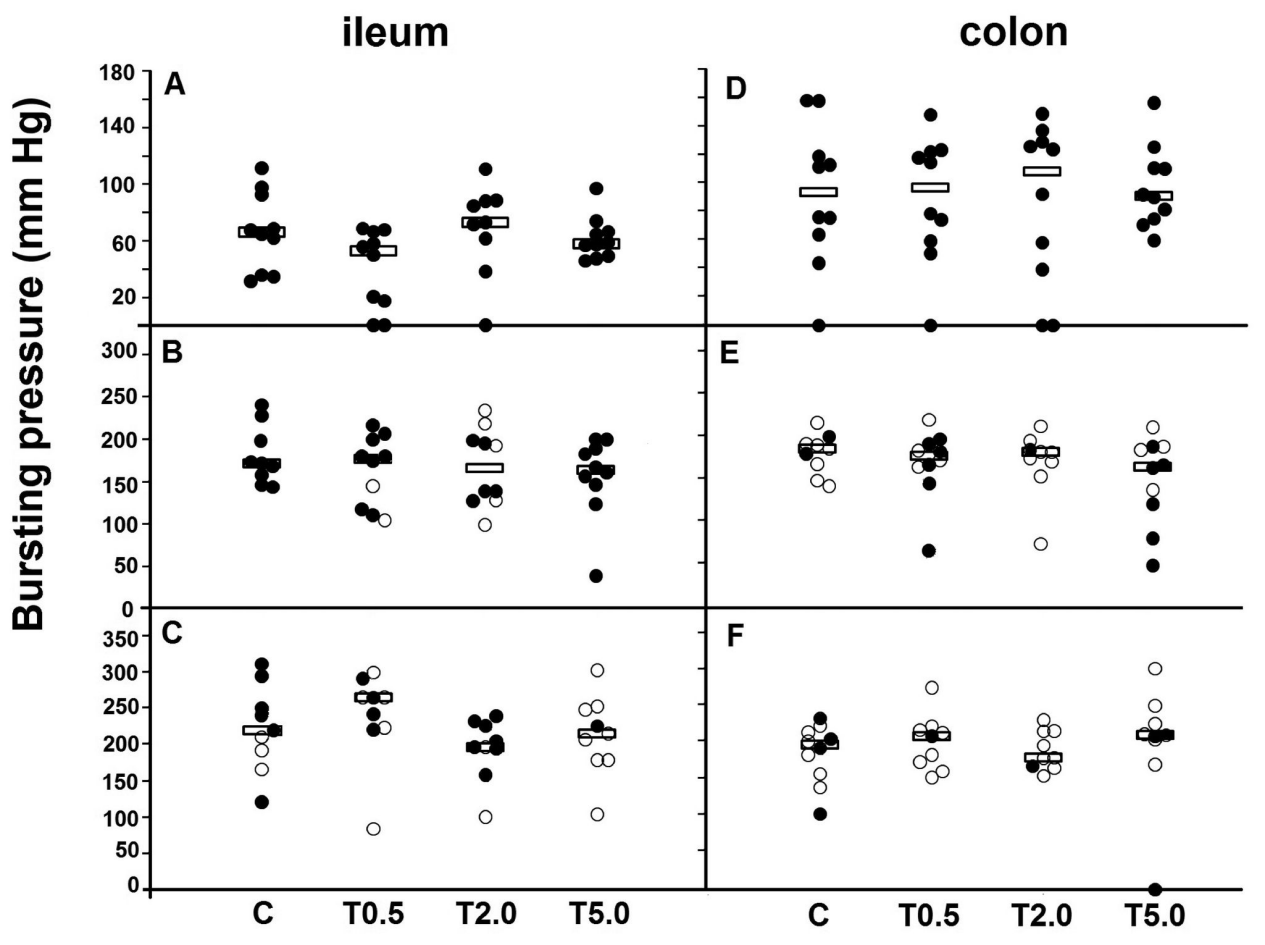
Anastomotic bursting pressure. Individual values and medians (horizontal lines) in ileum and colon. A,B,C = ileum 3,5 and 7 days postoperative. D,E,F = colon 3, 5 and 7 days postoperative. X-axis: study groups. Open symbols denote rupture outside the suture line and closed symbols rupture inside the suture line.

Individual values for anastomotic bursting pressures in the intestine are given in Figure 2. They increased with time from day 3 onwards and, at all time points, median values were comparable in all four groups. From day 5 the bursting site was increasingly frequent outside the true anastomotic area (Table 1). An increase in the number of anastomoses bursting outside the suture line represents an increase in anastomotic strength. Altogether, this phenomenon occurred equally frequent in control and tacrolimus groups.

**Table 1:** Anastomotic bursting site.

		**Ileum**	**Colon**	
	**C**	0/10	0/10	
Day 3	**T0.5**	0/10	0/10	
	**T2**	0/9	0/10	
	**T5**	1/10	0/10	
	**C**	0/9	7/9	
Day 5	**T0.5**	2/10	4/10	
	**T2**	5/10	8/9	
	**T5**	0/10	4/10	
	**C**	3/9	6/10	
Day 7	**T0.5**	5/9	8/9	
	**T2**	2/9	8/9	
	**T5**	8/9	6/9	

Numbers represent the frequency of the bursting site being without the actual suture line.

**Figure 3:**
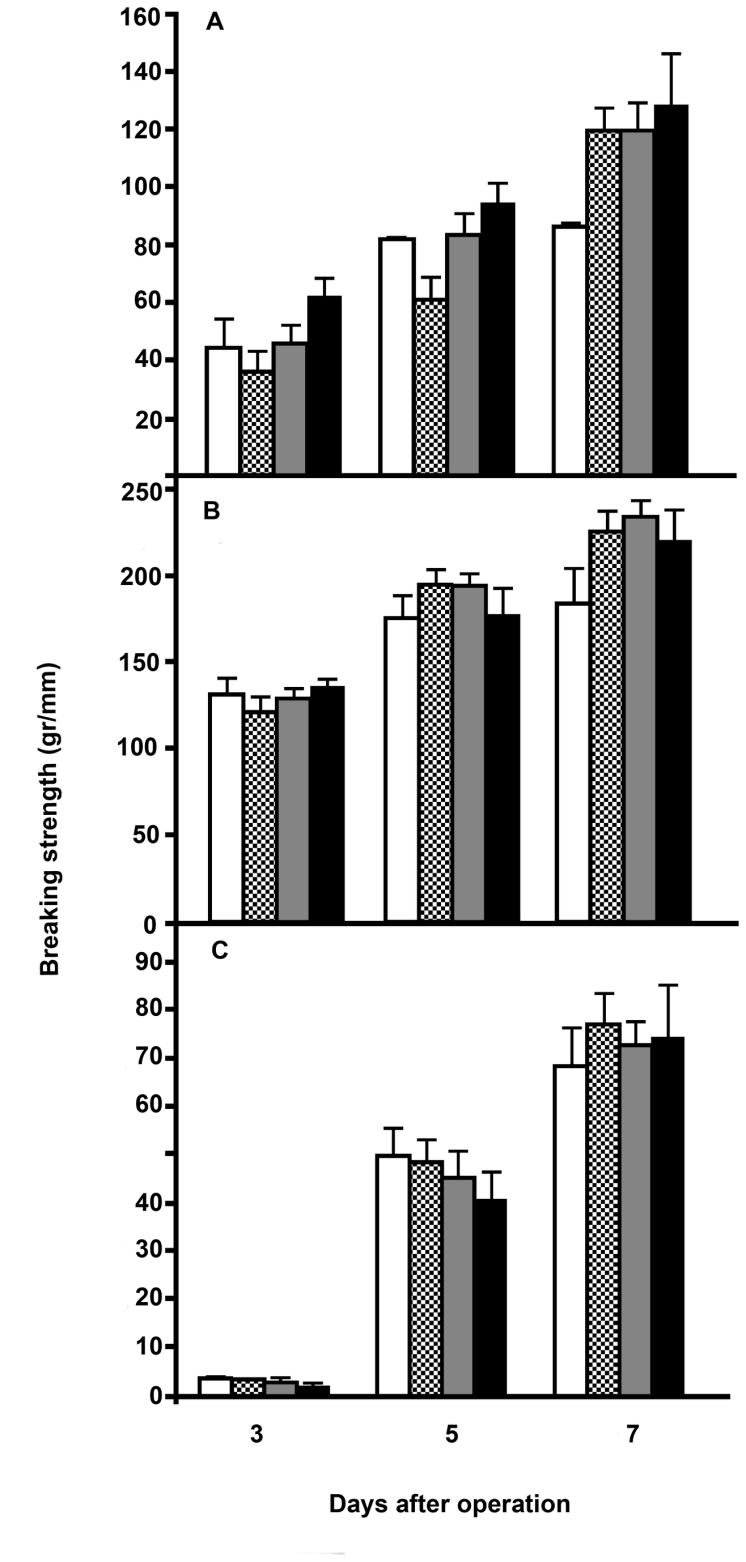
Wound breaking strength. Data represent mean and SEM in the control groups (white bars) and the T0.5 (black & white bars), T2 (grey bars) and T5 (black bars) tacrolimus groups. A= ileum anastomoses, B= colon anastomoses, C = fascia.

Wound breaking strength is depicted in Figure 3. After 3 days fascia strength was very low but increased rapidly thereafter. For all groups, the gain in fascia strength was very significant (comparison of values at 3, 5 and 7 days by ANOVA: p<0.0001) and similar. At no time there were significant differences between the four groups. For the intestinal anastomoses, the gain in strength between day 3 and day 5 was less explicit. Still, for all groups anastomotic strength increased significantly (p<0.05) with time, values at day 7 always being higher (Tukey-Kramer, p<0.05) than those at day 3. A significant difference between groups was only found for ileal anastomoses at day 7 where strength was higher in the T5 group than in the control group. Breaking always occurred within the suture line.

### Wound collagen content and gelatinase activity

**Figure 4:**
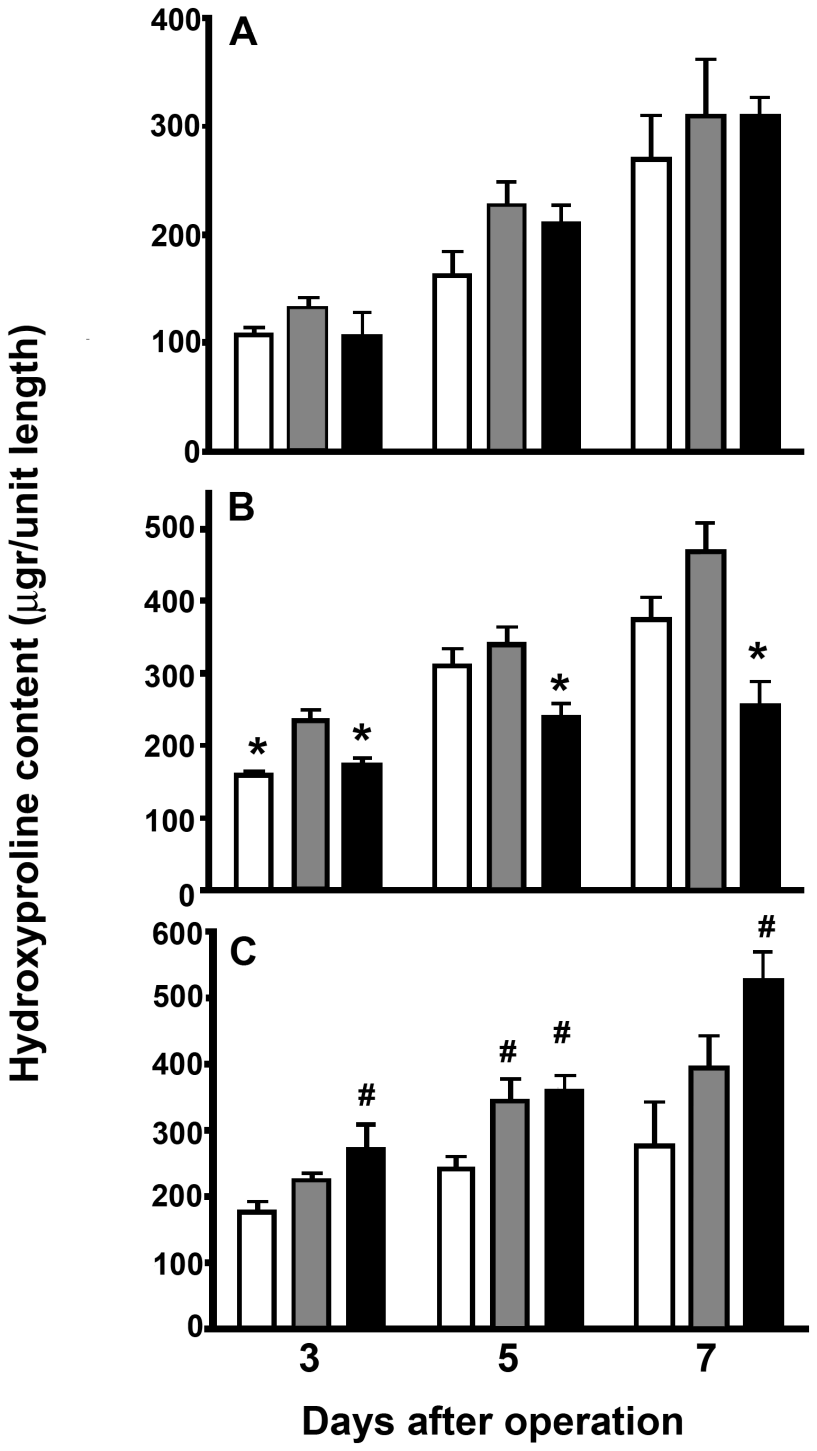
Wound hydroxyproline content. Data are expressed as hydroxyproline content per 5 mm tissue length and represent mean and SEM for the controls (white bars) and the T2 (grey bars) and T5 (black bars) tacrolimus groups. A= ileum, B= colon, C = fascia. *: p<0.05 vs T2 group; #: p<0.05 vs control group.

Wound hydroxyproline, as a measure for collagen content is given in Figure 4. Generally speaking, it increased with time, values at day 7 almost invariably being significantly (p<0.05) higher than those at day 3. In ileum, there were no differences between controls and experimental groups. In colon, values in the T5 group were lower than in the T2 group but equivalent to those in controls. In fascia, the hydroxyproline content was highest in the T5 groups.

**Figure 5:**
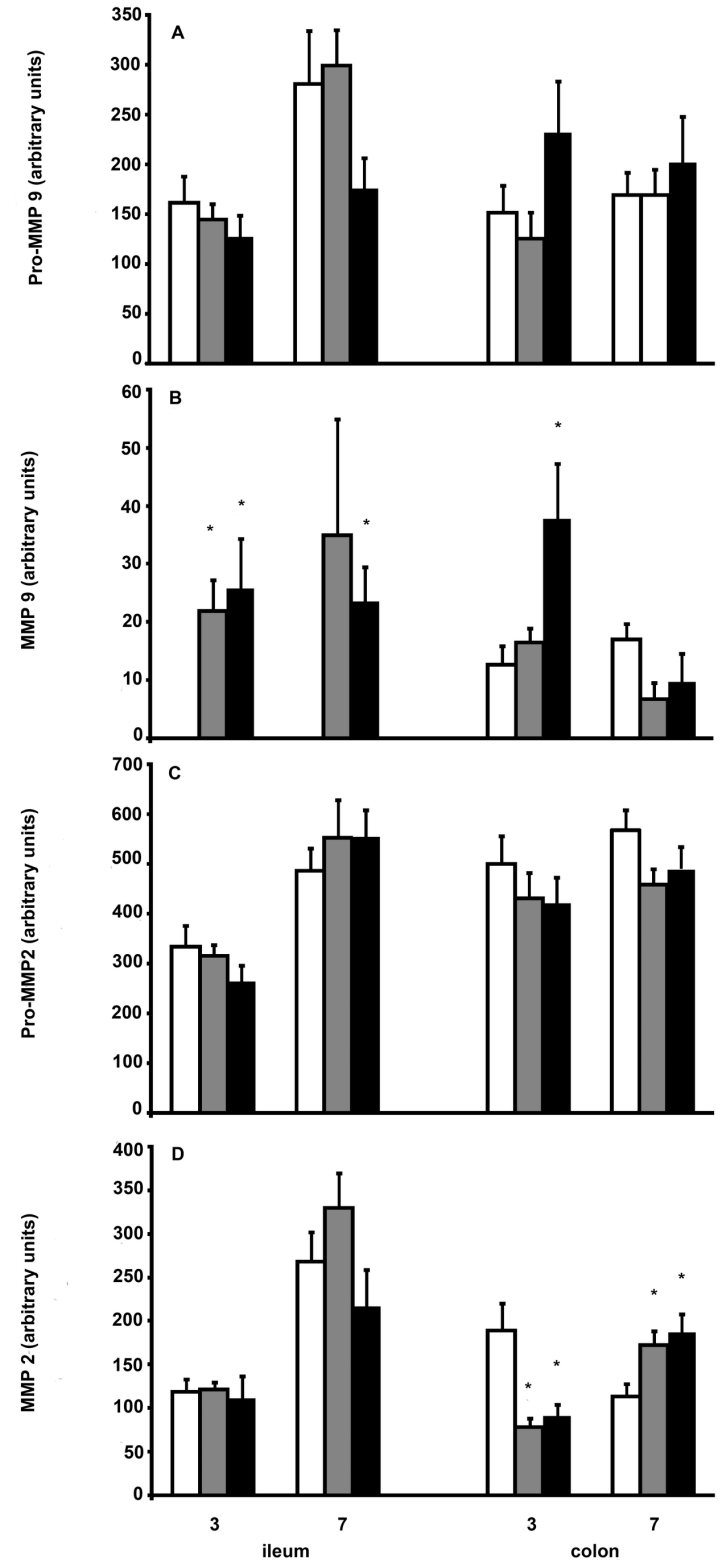
MMP activity in intestinal anastomoses. Columns represent mean values + SEM for controls (white bars) and the T2 (grey bars) and T5 (black bars) tacrolimus groups. Data represent total activities, in arbitrary units, per 5-mm segment for proMMP-9 (A), MMP-9 (B), proMMP-2 (C), and MMP-2 (D). *: p<0.05 vs control group.

The results of the zymographic measurements of gelatinase activity, in ileal and colonic tissue at day 3 and 7, are summarized in Figure 5. The activities of proMMP-9 and proMMP-2 were similar in control and tacrolimus groups. Although MMP-9 activities were low and varied considerably between animals, they appeared higher in the tacrolimus groups, especially in ileum. For MMP-2, differences were seen in colon where tacrolimus apparently lowered activity at day 3 while increasing it after 7 days.

### Histology

A comprehensive examination of sections obtained from controls and the T0.5 and T2 groups failed to reveal any obvious architectural differences at day 7. Semiquantitative analysis also failed to indicate any differences between control and the tacrolimus groups with respect to histologic parameters such as mucosal repair, epithelial damage, wound area surface, degree of necrosis, and cellular infiltration.

## Discussion

Although clinical studies of the effects on wound healing of single immunosuppressive drugs are lacking, wound complications after solid organ transplantation are often attributed to these drugs. Regimes including rapamycin derivates have been shown to be associated with more wound complications than those with calcineurin inhibitors. Our earlier experimental studies demonstrated a profound negative effect of everolimus on wound healing in the rat [6,7]. The present data clearly show that tacrolimus has no such effect and that surgical wounds demonstrate normal repair in the critical first week after operation, even during administration of doses leading to high trough levels of the drug. There is also no evidence for improvement of wound healing with tacrolimus.

Calcineurin inhibitors are notoriously known for their chronic toxicity and consequent chance of graft loss. Although cyclosporine and tacrolimus essentially inhibit the action of calcineurin in the same way, their side effects differ slightly. Tacrolimus is superior to cyclosporine in preventing acute rejection and improving graft survival which has resulted in an enormous increase in use of the agent since introduction in 1989 [10]. Tacrolimus has a greater effect on impairing the expression of alloantigen stimulated T-lymfocytes than does cyclosporine [19] but supposedly a less significant effect on wound healing. Clinically, it is always used together with other drugs, therefore, its safety in terms of interference with wound repair must be assessed in a preclinical model.

Preclinical data on the effects of tacrolimus on wound healing are few and contradictory. Doses of 2 mg/kg/day reportedly inhibit skin healing, but not colonic or ileal anastomotic healing [11,12,14,15]. Kiyama et al. even found that low doses (0.01-1 mg/kg/day) increased wound strength in the colon but not in the ileum [13]. In all these experimental studies wound repair was analyzed at one time point only. Raptis et al. also used a low tacrolimus dose of 0.1 mg/kg/day and reported evidence of enhanced rodent colonic anastomotic healing, measured as bursting pressure at 4 and 8 days postoperatively [16].

In order to obtain data which would allow a comprehensive analysis of the possible effects of tacrolimus on soft tissue repair we analyzed wounds in intestine and abdominal fascia. Tacrolimus was administered in three doses which, dose-dependently, led to trough levels ranging from 5 to 12 ng/ml. These levels are in the range often reported in clinical studies [20]. Finally, the study included three time points, covering the inflammatory and early proliferative phases of the healing sequence. The first week after operation is particularly important because wound strength remains low during the first few days and increases from day 3 onwards. Any interference in this period will enhance chances on wound dehiscence.

The results presented here are unambiguous. Tacrolimus, in three different clinically relevant dosages, does not interfere with and does not promote wound strength, which is the primary functional outcome parameter. This result holds for wounds in both small and large bowel and in the abdominal fascia at any of the three time points measured. For the bowel anastomoses, we measured two independent parameters for strength, the bursting pressure and the breaking strength. The bursting pressure, which represents the ability to withstand intraluminal pressure, only reflects wound strength if the bursting site is within the suture line which will not always be the case after day 3. Therefore, it is very relevant to collect additional data for the breaking strength, which reflects wound strength over the entire period analysed here. Interestingly, undisturbed healing proceeded despite the fact that the rats in the experimental groups were clearly in a more catabolic state than those in the control group (Figure 1).

The introduction of new immunosuppressant agents has increased graft survival. However, at the same time, long term complications not related to graft function, become increasingly important, much of them due to development of cardiovascular disease or malignancies, related to the use of immunosuppressant drugs. The proportion of deaths attributed to malignancy in the first decade after transplantation is as high as 26% and rising [21]. Presumably, this phenomenon will result in a rise in future operations not related to the transplanted organ. Surgery needs to be executed under immunosuppressant therapy, possibly with increased chances of wound complications such as bowel leakage or incisional hernia. The present data emphasize the fact that tacrolimus, contrary to other drugs used for immunosuppression, does not have a negative effect on wound healing. This may be of clinical consequence in the direct post-transplant phase, as well as in patients on immunosuppressant therapy that have to undergo surgical procedures for other reasons. Such knowledge, obtained from preclinical studies as the present one, is very relevant for determining an optimal immunosuppressant regime in terms of composition, timing and dosage.

## Conclusion

Tacrolimus, as a single drug, does not influence the repair sequence in soft tissues during the first week after operation.
